# Machine Learning-Based Prediction of Postoperative Deep Vein Thrombosis Following Tibial Fracture Surgery

**DOI:** 10.3390/diagnostics15141787

**Published:** 2025-07-16

**Authors:** Humam Baki, İsmail Bülent Özçelik

**Affiliations:** 1 Department of Orthopedics, Private Gaziosmanpaşa Hospital, Istanbul Yeni Yüzyıl University, 34245 Istanbul, Turkey; 2 El Istanbul Hand Surgery Microsurgery Group, İstanbul Yeni Yüzyıl University Gaziosmanpaşa Hospital Hand Surgery Unit, Faculty of Health Sciences, Nisantasi University, 34398 Istanbul, Turkey; ibozcelik@gmail.com

**Keywords:** deep vein thrombosis, machine learning, tibia fracture, orthopedic surgery

## Abstract

**Background/Objectives**: Postoperative deep vein thrombosis (DVT) is a common and serious complication after tibial fracture surgery. This study aimed to develop and evaluate machine learning (ML) models to predict the occurrence of DVT following tibia fracture surgery. **Methods**: A retrospective analysis was conducted on patients who had undergone surgery for isolated tibial fractures. A total of 42 predictive models were developed using combinations of six ML algorithms—logistic regression, support vector machine, random forest, extreme gradient boosting, Light Gradient Boosting Machine (LightGBM), and neural networks—and seven feature selection methods, including SHapley Additive exPlanations (SHAP), Least Absolute Shrinkage and Selection Operator (LASSO), Boruta, recursive feature elimination, univariate filtering, and full-variable inclusion. Model performance was assessed based on discrimination, quantified by the area under the receiver operating characteristic curve (AUC-ROC), and calibration, measured using Brier scores, with internal validation performed via bootstrapping. **Results**: Of 471 patients, 80 (17.0%) developed postoperative DVT. The ML models achieved high overall accuracy in predicting DVT. Twenty-four models showed similarly excellent discrimination (pairwise AUC comparisons, *p* > 0.05). The top-performing model (random forest with RFE) attained an AUC of ~0.99, while several others (including LightGBM and SVM-based models) also reached AUC values in the 0.97–0.99 range. Notably, support vector machine models paired with Boruta or LASSO feature selection demonstrated the best calibration (lowest Brier scores), indicating reliable risk estimation. The final selected SVM models achieved high specificity (≥95%) with moderate sensitivity (~75–80%) for DVT detection. **Conclusions**: ML models demonstrated high accuracy in predicting postoperative DVT following tibial fracture surgery. Support vector machine-based models showed particularly favorable discrimination and calibration. These results suggest the potential utility of ML-based risk stratification to guide individualized prophylaxis, warranting further validation in prospective clinical settings.

## 1. Introduction

Deep vein thrombosis (DVT) is a common and serious complication following orthopedic trauma surgery. In particular, patients with lower-extremity fractures (such as tibial fractures) face a substantial risk of postoperative DVT and its life-threatening sequela, pulmonary embolism (PE) [1,2]. DVT/PE events contribute significantly to postoperative morbidity and have been reported to be a leading cause of preventable in-hospital mortality in surgical patients [3]. Despite routine thromboprophylaxis, clinically important DVT still occurs in this population. Historical data indicate that in the absence of prophylaxis, DVT may develop in up to 50% of orthopedic trauma patients [4]. Standard thromboprophylaxis protocols in orthopedic trauma surgery commonly include pharmacological agents such as low-molecular-weight heparin (LMWH) or direct oral anticoagulants (DOACs), together with mechanical methods like intermittent pneumatic compression or graduated compression stockings [4,5]. Even with standard chemoprophylaxis protocols, appreciable residual DVT rates (in the order of 10–20% in various series) have been observed after fracture surgeries [5,6]. This persistent burden reflects the need for more effective risk assessment and preventive measures in orthopedic trauma settings.

Current approaches to DVT risk stratification in orthopedic surgery have important limitations. Conventional risk assessment models compress patient risk into broad categories using a limited set of factors. In practice, these models often lack granularity and may not perform well in orthopedic trauma patients. For example, a widely used generic risk score classifies essentially all major orthopedic surgery patients as high-risk, offering little ability to distinguish which fracture patients are at especially high versus moderate risk [7]. Moreover, traditional scoring systems do not account for the full complexity of DVT risk profiles. Orthopedic trauma patients frequently have numerous risk modifiers (e.g., advanced age, comorbid diseases, injury severity, immobility extent), each contributing incrementally to thrombosis risk [8].

Machine learning (ML) has emerged as a promising approach to address these challenges in risk prediction. ML algorithms can analyze high-dimensional clinical data and model complex, non-linear interactions among myriad risk factors, potentially yielding more accurate and personalized risk estimates than traditional methods. In diverse medical and surgical contexts, ML-based predictive models have outperformed conventional risk models in stratifying VTE risk [9]. Within orthopedic surgery, initial studies have demonstrated the potential of ML to improve DVT risk prediction. For example, ML-driven models in joint-arthroplasty patients have achieved higher discrimination for postoperative VTE events compared to older scoring tools [10]. In hospitalized trauma populations, combining a data-driven ML model with an existing risk assessment score significantly enhanced the identification of patients who went on to develop DVT [11]. These early findings suggest that ML can enable more nuanced and patient-specific risk stratification, which is particularly relevant for orthopedic trauma patients with variable profiles.

Recent work has increasingly explored machine learning approaches—including random forest, XGBoost, LightGBM, and Support Vector Machines—for individualized prediction of venous thromboembolism (VTE) risk in orthopedic surgery. In these studies, ML-based models have often outperformed conventional risk scores by incorporating a larger set of perioperative variables and modeling complex interactions. For example, Wang et al. examined lower-extremity fractures with XGBoost, while Huang et al. applied gradient boosting to national hip arthroplasty data [1,12]. Ge et al. demonstrated that LightGBM could effectively identify elderly hip fracture patients at increased risk [13]. Despite these advances, most prior work has focused on elective joint surgery or mixed trauma populations and has not systematically compared different feature selection methods alongside multiple ML algorithms. To address these gaps, the present study develops and benchmarks an extensive set of predictive models for DVT risk following tibial fracture surgery, with careful evaluation of both discrimination and calibration performance. This study aimed to develop and validate ML models for predicting postoperative DVT in patients undergoing tibial fracture surgery.

## 2. Materials and Methods

### 2.1. Study Design

This study was designed as a single-center, retrospective observational cohort involving patients who had undergone tibial fracture surgery at Istanbul Yeniyüzyıl University Hospital. The institutional ethics committee approved the study protocol (approval number: 2025/05-1542; date: 7 May 2025), and the need for individual informed consent was waived due to the retrospective design. All procedures were conducted in accordance with the Declaration of Helsinki.

### 2.2. Study Population

Eligible patients were identified using hospital electronic medical records and surgical operation logs. The study included adult individuals (aged 18 years or older) who had undergone isolated tibial fracture surgery between 1 January 2022 and 1 January 2025. To be included in the analysis, patients were required to have at least 30 days of postoperative follow-up and complete preoperative, perioperative, and laboratory data available in their clinical records. Patients were excluded if they had a documented diagnosis of deep vein thrombosis (DVT) prior to surgery, had undergone simultaneous bilateral lower-extremity procedures, or had tibial fractures associated with major vascular injury necessitating an alternative management approach. Cases with incomplete data for any key study variables were also excluded from the analysis.

### 2.3. Data Collection and Variable Definitions

Clinical data were retrospectively obtained from the hospital’s electronic health record system and archived patient charts using ICD-10 diagnostic and procedural codes related to tibial fracture surgery. For each eligible patient, a standardized data abstraction process was used to extract preoperative, perioperative, and postoperative variables. Demographic characteristics included age, sex, body mass index (BMI), and smoking status. Comorbidities were recorded based on documented medical history, including hypertension, diabetes mellitus, coronary artery disease, heart failure, chronic kidney disease, chronic pulmonary disease, and malignancy. The Charlson Comorbidity Index (CCI) was calculated to quantify overall disease burden. Preoperative clinical variables included admission vital signs—systolic and diastolic blood pressure, heart rate, body temperature, and oxygen saturation—as well as initial laboratory results, such as complete blood count (including white blood cell and lymphocyte counts), hemoglobin, platelet count, serum albumin, creatinine, coagulation parameters (prothrombin time, international normalized ratio, and activated partial thromboplastin time), D-dimer, and fibrinogen. Operative data comprised the surgical technique, fracture type (open or closed), Gustilo–Anderson classification for open fractures, anesthesia modality (general or regional), and time from injury to surgery. Postoperative variables included the timing of mobilization, the length of hospital stay, the administration and timing of thromboprophylaxis, and any complications such as infection, bleeding, or transfusion requirements. Delayed mobilization was defined as failure to ambulate within 48 h after surgery, and prolonged immobilization was defined as remaining bedridden for more than 7 days postoperatively.

All data were collected and verified by the research team using a uniform data collection form. Patient identities were anonymized by assigning study codes, and data consistency was cross-checked with the original records prior to statistical analysis.

### 2.4. Outcome Definition and Ascertainment

The primary outcome was the occurrence of postoperative DVT in the lower limb that was operated on, confirmed by compression Doppler ultrasonography during the follow-up period. All patients were monitored for clinical signs and symptoms of DVT for a minimum of 30 days following surgery. Outcome ascertainment was based on a review of inpatient and outpatient records, including clinical notes, specialist consultations, and discharge summaries. Imaging records were cross-checked through the hospital’s radiology system, and a diagnosis of DVT was accepted only when explicitly confirmed by Doppler ultrasound. In cases presenting symptoms after discharge, outpatient data and corresponding imaging reports were evaluated. Patients without documented DVT were considered outcome-negative. For patients with confirmed DVT, the date of diagnosis and any therapeutic interventions were recorded for analysis.

### 2.5. Machine Learning Selection

We selected six ML algorithms—logistic regression, support vector machine (SVM), random forest (RF), extreme gradient boosting (XGBoost), Light Gradient Boosting Machine (LightGBM), and neural networks—based on their successful application in orthopedic prediction tasks. Recent studies in orthopedic surgery have shown strong predictive performance of tree-based models like RF and XGBoost in tasks such as predicting nonhome discharge and complications following shoulder arthroplasty and red blood cell transfusions after orthopedic surgery [14,15]. Neural networks have also been shown to outperform classical models in orthopedic trauma prediction tasks, particularly for outcomes like mortality and postoperative length of stay [16]. The diversity of model architectures allows for robust benchmarking across varying data structures and prediction goals in orthopedic datasets. In our study, the neural network model was implemented as a multi-layer perceptron (MLP), selected for its proven performance in structured clinical datasets.

### 2.6. Feature Selection

To optimize model performance while maintaining interpretability, we employed a variety of feature selection methods. Recursive Feature Elimination (RFE) was particularly effective in orthopedic surgery datasets with high-dimensional clinical variables, as shown in a multicenter cohort study predicting blood transfusion risk [15]. SHAP values have also been increasingly applied in orthopedics to enhance model explainability, identifying key features, such as age, comorbidities, and surgical time, that contribute to outcome predictions [17]. LASSO, while traditionally used in general clinical settings, has also been applied in orthopedic studies due to its ability to produce parsimonious models, aiding making in robust, generalizable predictions [18]. By incorporating both regularization-based and model-specific selection techniques, our study maximized predictive efficiency and transparency.

### 2.7. Analysis

**Figure 1:**
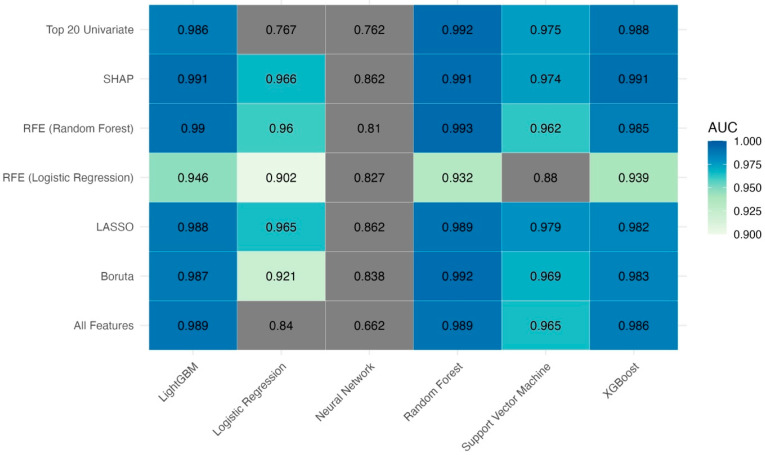
Heatmap of pairwise DeLong test *p*-values comparing AUCs of different machine learning models across feature selection methods. The heatmap illustrates the statistical significance of differences between model AUCs. Darker colors indicate stronger statistical differences.

All statistical analyses were performed using R version 4.4.2 (R Foundation for Statistical Computing, Vienna, Austria). The distribution of continuous variables was assessed visually using histograms. Normally distributed variables were reported as the mean ± standard deviation (SD), while non-normally distributed variables were summarized as the median [interquartile range, IQR]. Categorical variables were expressed as counts and percentages (*n* [%]). Between-group comparisons were conducted using independent samples *t*-tests or Mann–Whitney U tests for continuous variables, and Chi-square or Fisher’s exact tests for categorical variables, as appropriate. Two-sided *p*-values < 0.05 were considered statistically significant. Missing data were minimal across all variables; no parameter had more than 5% missingness. To address incomplete values while preserving statistical power, multiple imputation was applied using predictive mean matching via the mice package. Five imputed datasets were created and pooled according to Rubin’s rules. We developed machine learning models using six algorithms—logistic regression, support vector machine (SVM), random forest (RF), neural networks, extreme gradient boosting (XGBoost), and Light Gradient Boosting Machine (LightGBM)—using a 70/30 split of the dataset into training and test sets. Each model was paired with one of seven feature selection strategies—SHAP; LASSO; Boruta; recursive feature elimination (RFE) with either random forest or logistic regression; univariate filtering (*p* < 0.20); and the inclusion of all features—resulting in a total of 42 model–feature set combinations. The neural network model was implemented as a multi-layer perceptron (MLP) using the keras package in R. It consisted of two hidden layers with 64 and 32 units, respectively, each utilizing ReLU activation and dropout regularization (0.3 and 0.2). A sigmoid activation function was applied in the output layer for binary classification. The model was optimized with the Adam algorithm (learning rate = 0.001) and binary cross-entropy loss. Training was conducted for 100 epochs with early stopping based on validation loss. Model discrimination was evaluated using the area under the receiver operating characteristic curve (AUC), with 95% confidence intervals (CI) estimated via 1000 bootstrap replicates. Pairwise comparisons of AUCs were conducted using the DeLong test. Figure 1 presents a heatmap of AUC comparisons, and models not statistically different from the top performers (*p* > 0.05) were retained for further evaluation. Calibration performance was assessed using Brier scores, with 95% CIs calculated from 1000 bootstrap replicates. Pairwise comparisons of Brier scores between models were performed using Wilcoxon signed-rank tests to identify robust and well-calibrated models. For the four best-performing models, diagnostic performance metrics—sensitivity, specificity, accuracy, and F1 score—were calculated along with 95% CIs using nonparametric bootstrapping. To enhance model interpretability, permutation-based feature importance analysis was performed using the iml package, with log-loss as the evaluation metric. Feature importances were normalized within each model, and the top five predictors were visualized.

## 3. Results

A total of 471 patients who underwent isolated tibia fracture surgery were included, of whom 80 (17.0%) developed postoperative deep vein thrombosis (DVT). The baseline demographic and comorbidity characteristics are presented in Table 1. Patients in the DVT group were significantly older (68.2 ± 8.8 vs. 55.3 ± 13.3 years, *p* < 0.001), more frequently female (55.0% vs. 39.1%, *p* = 0.013), and had higher rates of cardiovascular disease (33.8% vs. 16.6%, *p* < 0.001), chronic kidney disease (22.5% vs. 5.4%, *p* < 0.001), and COPD (17.5% vs. 8.4%, *p* = 0.024). They also demonstrated significantly lower albumin levels (2.75 [2.50–2.92] vs. 3.03 [2.74–3.27] g/dL, *p* < 0.001), reduced lymphocyte counts (2.07 ± 0.44 vs. 2.36 ± 0.46, *p* < 0.001), and elevated D-dimer and leukocyte values.

The operative and postoperative characteristics are summarized in Table 2. Compared to those without DVT, affected patients had longer operative times (134.2 ± 29.8 vs. 101.8 ± 23.8 min, *p* < 0.001), had higher rates of general anesthesia (75.0% vs. 54.0%, *p* = 0.002), and were more likely to present with open fractures and higher Gustilo grades (both *p* < 0.001). Delays in mobilization and preoperative immobilization were also more frequent (both *p* < 0.001). Notably, postoperative CRP levels were substantially elevated in the DVT group (74.4 ± 21.1 vs. 39.7 ± 21.7 mg/L, *p* < 0.001).

To predict postoperative DVT, we evaluated 42 machine learning models generated from combinations of six feature selection strategies and seven classifiers. Model discrimination was assessed using the AUC, and pairwise statistical comparisons of the 95% confidence intervals were performed using the DeLong test. These results are visualized in Figure 1. Among the 42 models, the top 24 models demonstrated no statistically significant differences from each other in terms of AUC (*p* > 0.05), indicating similar discrimination performance (Table 3). The best-performing model was random forest + RFE (AUC: 0.9948; 95% CI: 0.9875–1.0000), followed closely by the SHAP- and Boruta-based random forest and LightGBM models. SVM models, while slightly lower in terms of AUC (range: 0.969–0.978), remained competitive.

**Figure 2:**
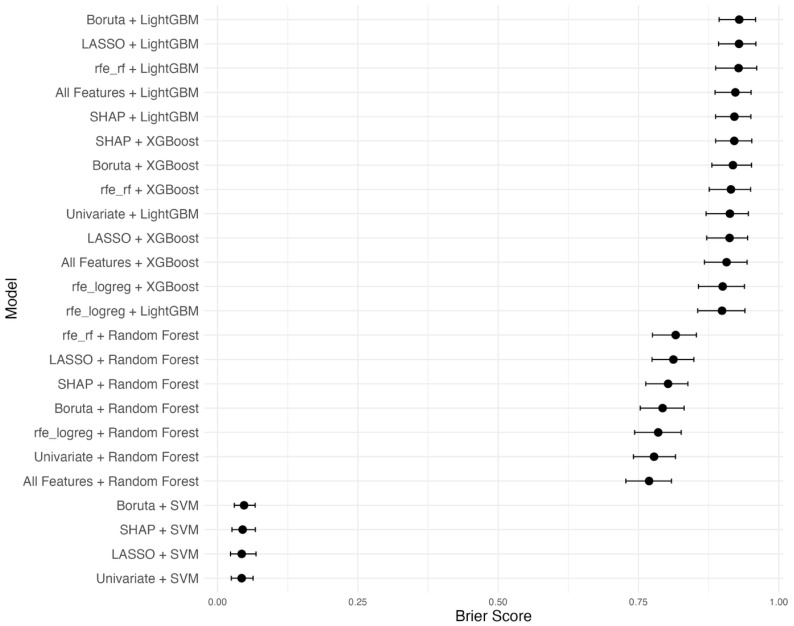
Bootstrapped Brier scores with 95% confidence intervals for various machine learning models and feature selection combinations. Models are ordered by mean Brier score, with lower scores indicating better predictive calibration.

Model calibration performance was assessed using the Brier score and its bootstrapped 95% CI (Figure 2). The comparison of Brier scores among the top 24 models revealed that SVM classifiers paired with Boruta, LASSO, or univariate feature selection achieved the lowest and most stable Brier scores, reflecting superior calibration. These four SVM models were thus selected for further evaluation.

Their diagnostic performance metrics are shown in Table 4. All models demonstrated high specificity (≥0.95) and good overall accuracy. Boruta + SVM achieved the highest sensitivity (0.79; 95% CI: 0.61–0.95), while both SHAP + SVM and LASSO + SVM achieved the highest F1 scores (0.78).

In addition to discrimination and calibration performance, detailed diagnostic metrics were calculated for the four selected SVM models. Supplementary Table S1 presents the precision, recall, specificity, PPV, NPV, and log-loss with corresponding 95% confidence intervals. All models demonstrated high negative predictive value (NPV: 0.961–0.968) and specificity (0.953–0.977), with precision and PPV ranging between 0.714 and 0.824. Log-loss values were comparable across models, ranging from 0.1384 to 0.1548.

**Figure 3:**
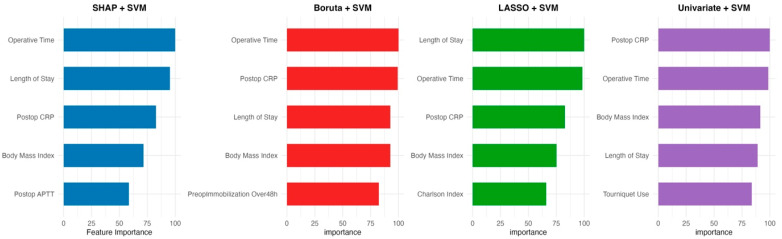
The top 5 most important features across the four SVM models using different feature selection strategies. Each barplot displays the top five predictors contributing most to the model performance, based on permutation-based feature importance measured with log-loss increase. The feature importance values were normalized within each model (maximum = 100%).

Lastly, Figure 3 displays the top five most important features for each of the selected SVM models, based on permutation-based feature importance using log-loss increase. Operative time, postoperative CRP, and length of stay were consistently important across models. Tourniquet use and the Charlson Comorbidity Index were selectively prominent in the univariate and LASSO-based SVM models, respectively, highlighting differences in feature reliance across selection strategies.

## 4. Discussion

The present study demonstrates that ML can effectively stratify postoperative DVT risk in patients undergoing tibial fracture surgery. Using a comprehensive modeling approach, we found that several ML models—particularly those based on support vector machines—achieved strong predictive performance. Our top models showed excellent discrimination and robust calibration, suggesting that individualized risk prediction for DVT is feasible with preoperative and perioperative variables. Importantly, the models identified clinically plausible risk factors (such as longer operative duration and elevated inflammatory markers) as key contributors to DVT risk, aligning with known thrombogenic mechanisms.

Postoperative DVT remains a critical concern in lower-extremity orthopedic surgery. It is a major preventable cause of morbidity and mortality in hospitalized patients [19]. Even with routine thromboprophylaxis, orthopedic trauma patients face substantial residual risk. For instance, reported DVT incidences after fracture surgery can range from ~2% in lower-risk tibial fractures up to 16–32% in more severe femoral or pelvic fractures despite prophylaxis. In hip fracture surgery patients, early ultrasound surveillance has shown that approximately 24% develop DVT even under standard anticoagulant prophylaxis [20,21]. These findings emphasize the clinical relevance of thorough DVT risk assessment in orthopedic practice, as undiagnosed cases may result in life-threatening pulmonary embolism or long-term complications such as post-thrombotic syndrome. Improving risk stratification strategies may support more individualized prophylactic approaches in surgically vulnerable populations.

The present study contributes to a growing body of literature applying ML techniques to predict venous thromboembolism in surgical patients. Similar high-performance models have been reported in orthopedic cohorts. In patients with lower-extremity fractures, Wei et al. achieved excellent discrimination, with tree-based algorithms (XGBoost AUC ≈ 0.98) outperforming logistic regression and SVM [22]. Likewise, in elective arthroplasty, ML models have shown strong predictive accuracy. Huang et al. leveraged a national surgical dataset for total hip arthroplasty and reported an AUC of ~0.93 using a gradient boosting model, with calibration slopes near unity and low prediction bias [12]. Another recent study in elderly hip fracture patients (with comorbid hypertension) compared multiple algorithms and found that a LightGBM model yielded an AUC around 0.90 on external testing, alongside favorable decision-curve net benefit and calibration profiles [13]. These examples mirror our results in demonstrating that modern ML—from ensemble methods to SVMs—can substantially outperform traditional risk scores in identifying patients at risk for postoperative DVT.

Not all studies have reported such high discrimination, suggesting that patient population and feature selection play a role. For instance, in a large cohort of spine fusion surgery patients, Heo et al. noted more modest performance (best AUC of ~0.68 with a linear SVM) for 90-day VTE prediction, likely reflecting the lower event rate and different risk profile in that setting. Conversely, some specialized contexts have found certain algorithms particularly effective: in rehabilitation inpatients, an artificial neural network was the top performer for DVT prediction, with prolonged immobilization (bedrest duration) and D-dimer levels emerging as dominant predictors [23,24]. Across the literature, there is convergence on several risk factors—advanced age, delayed mobilization, elevated coagulopathy markers (e.g., D-dimer), and medical comorbidities (like cancer or prior VTE) are repeatedly identified as significant contributors to postoperative DVT risk [12,24].

The feature importance findings from this study—emphasizing variables such as operative time, postoperative CRP, tourniquet use, and the Charlson Comorbidity Index—align with established pathophysiological mechanisms, including venous stasis, tissue trauma, and inflammation-driven hypercoagulability. Taken together with findings from recent ML-based research, the present analysis supports the utility of ML in enhancing DVT risk stratification in orthopedic surgery. These models, by processing complex clinical data, offer the opportunity to identify high-risk patients for individualized prophylaxis and postoperative monitoring, advancing toward more personalized thrombosis prevention strategies.

### Limitations

This study has certain limitations that should be acknowledged. Its retrospective, single-center design may limit the generalizability of the findings, as institutional practices and patient populations may differ across settings. Although the overall sample size was moderate, the number of DVT events was relatively limited for ML analysis, which may affect the stability of some models despite efforts to mitigate overfitting through appropriate validation strategies. The analysis focused on perioperative variables available during hospitalization, without capturing longer-term or behavioral factors—such as rehabilitation adherence—that may also influence thrombotic risk. All patients received standardized thromboprophylaxis, which restricts the model’s ability to account for variations in prophylactic regimens. The tool developed in this study is intended to support, rather than replace, clinical decision-making and does not incorporate patient-specific considerations such as bleeding risk or treatment preferences. Finally, the study did not assess the clinical impact of applying the model in real time. In addition, because the models were trained on a specific orthopedic cohort (isolated tibial fracture surgery patients), their direct applicability to other surgical procedures remains uncertain without external validation and retraining. Future prospective studies are necessary to evaluate whether ML-guided prophylaxis strategies can improve patient outcomes.

## 5. Conclusions

This study demonstrated that ML models, particularly those based on SVM, can accurately predict postoperative DVT risk following tibial fracture surgery. These findings support the potential utility of ML-based risk stratification in guiding individualized thromboprophylaxis. Further external validation and prospective evaluation are needed before the clinical implementation of these models.

## Figures and Tables

**Table 1:** Baseline demographic, comorbidity, and preoperative characteristics of patients with prior tibia fracture surgery by DVT status.

Variable	No DVT (*n* = 391)	DVT (*n* = 80)	*p*
Age, years	55.3 ± 13.3	68.2 ± 8.8	<0.001
Sex, female	153 (39.1%)	44 (55.0%)	0.013
BMI	26.7 ± 4.1	29.3 ± 5.1	<0.001
Smoking—never	225 (57.5%)	29 (36.2%)	
Smoking—former	90 (23.0%)	21 (26.2%)	
Smoking—current	76 (19.4%)	30 (37.5%)	<0.001
History of VTE	9 (2.3%)	4 (5.0%)	0.250
Active malignancy	12 (3.1%)	5 (6.2%)	0.289
Cardiovascular disease	65 (16.6%)	27 (33.8%)	<0.001
Diabetes mellitus	71 (18.2%)	21 (26.2%)	0.131
Chronic kidney disease	21 (5.4%)	18 (22.5%)	<0.001
COPD	33 (8.4%)	14 (17.5%)	0.024
Charlson Comorbidity Index	2 [1–3]	3 [2–4]	<0.001
Pre-injury mobility—Independent	212 (54.2%)	19 (23.8%)	
Pre-injury mobility—Assisted	169 (43.2%)	52 (65.0%)	
Pre-injury mobility—Bedridden	10 (2.6%)	9 (11.2%)	<0.001
Albumin, g/dL	3.03 [2.74–3.27]	2.75 [2.50–2.92]	<0.001
Lymphocyte count	2.36 ± 0.46	2.07 ± 0.44	<0.001
Hemoglobin, g/dL	12.9 ± 0.8	12.8 ± 0.84	0.387
D-dimer (preop), mg/L	1.4 [0.85–2.4]	1.8 [1.08–2.83]	0.010
APTT (preop), s	29.4 ± 1.03	29.8 ± 1.09	0.002
Fibrinogen, mg/dL	421.0 ± 56.5	436.5 ± 48.9	0.013
Leukocyte count	9.36 ± 1.33	10.0 ± 1.45	<0.001
Platelet count	255.7 ± 30.0	254.6 ± 29.7	0.763

DVT, deep vein thrombosis; VTE, venous thromboembolism; COPD, chronic obstructive pulmonary disease; CCI, Charlson Comorbidity Index; APTT, activated partial thromboplastin time; SD, standard deviation; IQR, interquartile range.

**Table 2:** Operative and postoperative characteristics of patients with prior tibia fracture surgery by deep vein thrombosis status.

Variable	No DVT (*n* = 391)	DVT (*n* = 80)	*p*	Δ Mean (95% CI)
Operative time, min	101.8 ± 23.8	134.2 ± 29.8	<0.001	32.4 (25.6–39.2)
Tourniquet use	344 (88.0%)	50 (62.5%)	<0.001	
Blood loss, mL	238.8 ± 97.1	264.2 ± 115.3	0.068	
Anesthesia—spinal	211 (54.0%)	60 (75.0%)	0.002	
Anesthesia—general	141 (36.1%)	15 (18.8%)		
Anesthesia—combined	39 (10.0%)	5 (6.2%)		
Implant—IM nail	246 (62.9%)	41 (51.2%)	0.002	
Implant—plate	119 (30.4%)	24 (30.0%)		
Implant—external fixator	26 (6.6%)	15 (18.8%)		
Fracture—proximal	207 (52.9%)	34 (42.5%)	0.015	
Fracture—diaphyseal	121 (30.9%)	38 (47.5%)		
Fracture—distal	63 (16.1%)	8 (10.0%)		
Open fracture	80 (20.5%)	32 (40.0%)	<0.001	
Gustilo—closed	281 (71.9%)	46 (57.5%)	<0.001	
Gustilo—Type I	77 (19.7%)	14 (17.5%)		
Gustilo—Type II	22 (5.6%)	15 (18.8%)		
Gustilo—Type III	11 (2.8%)	5 (6.2%)		
Injury to surgery, days	2.05 ± 1.08	2.69 ± 1.16	<0.001	0.64 (0.37–0.91)
Postop immobilization > 7 d	71 (18.2%)	47 (58.8%)	<0.001	
Mobilization delay > 48 h	100 (25.6%)	65 (81.2%)	<0.001	
Preop immobilization > 48 h	66 (16.9%)	52 (65.0%)	<0.001	
Any postop complication	88 (22.5%)	53 (66.2%)	<0.001	
Blood transfusion	91 (23.3%)	27 (33.8%)	0.067	
CRP (postop), mg/L	39.7 ± 21.7	74.4 ± 21.1	<0.001	34.7 (29.6–39.8)
ESR (postop), mm/h	23.4 ± 11.1	25.1 ± 10.1	0.199	
APTT (postop), s	35.9 ± 0.53	36.2 ± 0.58	<0.001	0.3 (0.16–0.44)
Disturbance of consciousness	26 (6.6%)	21 (26.2%)	<0.001	
Vasoactive drug use	29 (7.4%)	18 (22.5%)	<0.001	
Length of stay, days	8 [6–9]	11 [10–12]	<0.001	

DVT, deep vein thrombosis; CRP, C-reactive protein; ESR, erythrocyte sedimentation rate; APTT, activated partial thromboplastin time; IQR, interquartile range; SD, standard deviation.

**Table 3:** Ranked performance of robust models by AUC and 95% confidence intervals.

Rank	Feature Selection Method	Model Type	AUC (95% CI)
1	RFE (RF)	RF	0.9948 (0.9875–1.0000)
2	SHAP	RF	0.9936 (0.9848–1.0000)
3	All Features	RF	0.9925 (0.9833–1.0000)
4	Univariate (*p* < 0.20)	RF	0.9913 (0.9809–1.0000)
5	SHAP	LGBM	0.9905 (0.9789–1.0000)
6	SHAP	XGB	0.9905 (0.9791–1.0000)
7	RFE (RF)	LGBM	0.9901 (0.9786–1.0000)
8	All Features	LGBM	0.9888 (0.9750–1.0000)
9	LASSO	LGBM	0.9876 (0.9735–1.0000)
10	Boruta	RF	0.9872 (0.9728–1.0000)
11	LASSO	RF	0.9872 (0.9717–1.0000)
12	Boruta	LGBM	0.9867 (0.9704–1.0000)
13	Univariate (*p* < 0.20)	LGBM	0.9859 (0.9700–1.0000)
14	All Features	XGB	0.9855 (0.9697–1.0000)
15	RFE (RF)	XGB	0.9847 (0.9692–1.0000)
16	Boruta	XGB	0.9834 (0.9633–1.0000)
17	LASSO	XGB	0.9822 (0.9644–0.9999)
18	LASSO	SVM	0.9785 (0.9580–0.9989)
19	Univariate (*p* < 0.20)	SVM	0.9747 (0.9524–0.9971)
20	SHAP	SVM	0.9743 (0.9513–0.9973)
21	Boruta	SVM	0.9689 (0.9399–0.9979)
22	RFE (LogReg)	LGBM	0.9461 (0.8890–1.0000)
23	RFE (LogReg)	XGB	0.9387 (0.8677–1.0000)
24	RFE (LogReg)	RF	0.9128 (0.8184–1.0000)

RFE = recursive feature elimination; RF = random forest; LGBM = Light Gradient Boosting Machine; XGB = XGBoost; SVM = support vector machine; LogReg = logistic regression; SHAP = SHapley Additive exPlanations.

**Table 4:** Diagnostic performance of selected SVM models.

Model	Sensitivity (95% CI)	Specificity (95% CI)	Accuracy (95% CI)	F1 Score (95% CI)
Boruta + SVM	0.79 (0.61–0.95)	0.95 (0.91–0.98)	0.93 (0.89–0.97)	0.75 (0.59–0.87)
LASSO + SVM	0.74 (0.53–0.92)	0.98 (0.95–1.00)	0.95 (0.90–0.98)	0.78 (0.61–0.91)
SHAP + SVM	0.74 (0.53–0.92)	0.98 (0.95–1.00)	0.95 (0.90–0.98)	0.78 (0.61–0.91)
Univariate + SVM	0.74 (0.53–0.93)	0.96 (0.93–0.99)	0.93 (0.89–0.97)	0.73 (0.55–0.88)
LASSO + SVM	0.74 (0.53–0.92)	0.98 (0.95–1.00)	0.95 (0.90–0.98)	0.78 (0.61–0.91)
SHAP + SVM	0.74 (0.53–0.92)	0.98 (0.95–1.00)	0.95 (0.90–0.98)	0.78 (0.61–0.91)
Univariate + SVM	0.74 (0.53–0.93)	0.96 (0.93–0.99)	0.93 (0.89–0.97)	0.73 (0.55–0.88)

SVM, support vector machine; LASSO, Least Absolute Shrinkage and Selection Operator; SHAP, SHapley Additive exPlanations.

## Data Availability

The data that support the findings of this study are available from the corresponding author upon reasonable request.

## Supplementary Materials

The following are available online at https://www.mdpi.com/article/10.3390/diagnostics15141787/s1, Table S1: Supplementary Performance Metrics for Selected Support Vector Machine Models (95% CI).

## Abbreviations

The following abbreviations are used in this manuscript:

APTT	Activated Partial Thromboplastin Time
AUC	Area Under the Curve
BMI	Body Mass Index
CCI	Charlson Comorbidity Index
CI	Confidence Interval
COPD	Chronic Obstructive Pulmonary Disease
CRP	C-Reactive Protein
DVT	Deep Vein Thrombosis
ICD-10	International Classification of Diseases, 10th Revision
IQR	Interquartile Range
LASSO	Least Absolute Shrinkage and Selection Operator
LightGBM	Light Gradient Boosting Machine
LogReg	Logistic Regression
ML	Machine Learning
PE	Pulmonary Embolism
RFE	Recursive Feature Elimination
RF	Random Forest
SD	Standard Deviation
SHAP	SHapley Additive exPlanations
SVM	Support Vector Machine
VTE	Venous Thromboembolism
XGBoost	Extreme Gradient Boosting
