# Supplementary material for: Machine Learning-Based Prediction of Postoperative Deep Vein Thrombosis Following Tibial Fracture Surgery
Source: Diagnostics (Basel). 2025 Jul 16;15(14):1787. doi: 10.3390/diagnostics15141787 (PMC12293441; doi:10.3390/diagnostics15141787)
Supplement: Supplementary file 1 [file diagnostics-15-01787-s001.zip › diagnostics-3715619-Supplementary.pdf]

Supplementary Table S1. Supplementary Performance Metrics for Selected Support Vector Machine Models (95% CI)

Model	Precision (95% CI)	Recall (95% CI)	Specificity (95% CI)	PPV (95% CI)	NPV (95% CI)	Log-loss (95% CI)
Boruta + SVM	0.714 (0.500–0.909)	0.789 (0.588–0.952)	0.953 (0.918–0.981)	0.714 (0.500–0.909)	0.968 (0.931–0.992)	0.1532 (0.1023–0.2121)
LASSO + SVM	0.818 (0.600–1.000)	0.733 (0.500–0.923)	0.976 (0.949–1.000)	0.822 (0.615–1.000)	0.961 (0.925–0.992)	0.1536 (0.0985–0.2178)
SHAP + SVM	0.824 (0.615–1.000)	0.737 (0.534–0.913)	0.977 (0.947–1.000)	0.822 (0.625–1.000)	0.962 (0.923–0.992)	0.1548 (0.1054–0.2049)
Univariate + SVM	0.732 (0.526–0.923)	0.739 (0.526–0.933)	0.960 (0.921–0.992)	0.742 (0.526–0.929)	0.961 (0.921–0.992)	0.1384 (0.0899–0.1897)
Model	Precision (95% CI)	Recall (95% CI)	Specificity (95% CI)	PPV (95% CI)	NPV (95% CI)	Log-loss (95% CI)
Boruta + SVM	0.714 (0.500–0.909)	0.789 (0.588–0.952)	0.953 (0.918–0.981)	0.714 (0.500–0.909)	0.968 (0.931–0.992)	0.1532 (0.1023–0.2121)
LASSO + SVM	0.818 (0.600–1.000)	0.733 (0.500–0.923)	0.976 (0.949–1.000)	0.822 (0.615–1.000)	0.961 (0.925–0.992)	0.1536 (0.0985–0.2178)

PPV: Positive Predictive Value; NPV: Negative Predictive Value.
